# Practical steps needed to achieve impact of the WHO 2019 movement behaviour guidelines for children under the age of 5: the SUNRISE Study Europe Group evaluation

**DOI:** 10.1016/j.lanepe.2024.100869

**Published:** 2024-02-28

**Authors:** Marieke De Craemer, Sanne L.C. Veldman, Liane B. Azevedo, Farid Bardid, Jesus Del Pozo Cruz, Elina Engberg, Juel Jarani, Anna Kontsevaya, Marie Löf, Clarice Martins, Hanna Nalecz, Anthony Okely, Mark Tremblay, Fotini Venetsanou, Mehmet Yildiz, John J. Reilly

**Affiliations:** a Department of Rehabilitation Sciences, Ghent University, Corneel Heymanslaan 10, Ghent, Belgium; b Mulier Institute, Utrecht, Netherlands; c Sheffield Hallam University, Advanced Wellbeing Research Centre, 2 Old Hall Rd, Sheffield, S93TU, England; d University of Strathclyde, Strathclyde Institute of Education, 16 Richmond Street, Glasgow, G1 1XQ, Scotland; e Departamento de Educación Fisica y Deporte, Universidad de Sevilla, Sevilla, Spain; f Folkhälsan Research Center, Topeliuksenkatu 20, 00250, Helsinki, Finland; g Albanian Sports Science Association, Albania; h National Research Center for Preventive Medicine, Ministry of Healthcare, Russian Federation; i Department of Biosciences and Nutrition, Karolinska Institute, NEO/Group MLÖ, 141 83 Huddinge, Stockholm, Sweden; j Research Centre of Physical Activity, Health and Leisure, Faculty of Sports, Laboratory for Integrative and Translational Research in Population Health (ITR), University of Porto, 4500, Porto, Portugal; k Jozef Pilsudski University of Physical Education, Faculty of Physical Education, ul. Marymoncka 34, 00-968, Warsaw, Poland; l School of Health and Society, University of Wollongong, Australia and Department of Sport, Food and Natural Sciences, Western Norway University of Applied Sciences, Sogndal Norway; m Healthy Active Living and Obesity Research Group, Children's Hospital of Eastern Ontario Research Institute, Ottawa, Canada; n School of Physical Education and Sport Science, National and Kapodistrian University of Athens, Greece; o Faculty of Sports Science, Afyon Kocatepe University, Turkiye; p Department of Psychological Sciences and Health, University of Strathclyde, 16 Richmond Street, Glasgow, G1 1XQ, Scotland; q School of Health and Society, Faculty of the Arts, Social Sciences and Humanities, University of Wollongong, Australia

The World Health Organisation (WHO) guidelines for the ‘24-h movement behaviours’^1^ (physical activity (PA), sedentary behaviour (SB) including screen time, and sleep) in the under-5s were published in April 2019 (Supplementary Figure S1).^2^ The guidelines were developed as a response to the childhood obesity pandemic,^2^ to help ensure that under-5s have healthy levels of PA, screen time, and sleep. Evidence review and synthesis showed that these behaviours influenced a wide range of other outcomes, with substantial short-term and long-term consequences (e.g., cognitive, social and emotional development; language development; cardiometabolic health; bone and skeletal health; motor development; physical fitness; growth; and wellbeing).^2^

Five years later, it is now appropriate to test whether key actions in response to these guidelines were taken across Europe, and to consider ways of increasing the impact of the WHO Guidelines across Europe in the next 5 years. Therefore, the SUNRISE Study Europe Group considered three tests to examine if European public health policy and clinical practice were sufficiently responsive to the WHO Guidelines: (1) Do European nations have national guidelines for the movement behaviours in the under-5s or have they adopted/adapted the WHO Guidelines? (2) Do they have adequate surveillance of the movement behaviours in these age groups? (3) Do they have specific movement behaviour policies for children under-5?

European countries have largely failed to create national guidelines or adopt/adapt the WHO guidelines (Test 1), to organise surveillance of the movement behaviours (Test 2) and create national health policy for all movement behaviours for the under-5s (Test 3) (Box 1). This means that the opportunity for public health gain following the release of the WHO guidelines has not yet been realised.^2^ The failure of tests 1–3 is also a concern because of recent evidence from research studies on meeting the guidelines. A recent pooled analysis (manuscript in preparation) found that ±75% of European preschoolers did not meet the combined guidelines prior to the COVID-19 pandemic.^3^ COVID-19 mitigation measures generally reduced PA and increased screen time.^4^ The prevalence of not meeting the guidelines is likely to be even higher in some population sub-groups such as those with chronic disease and/or disability.^5^ Lack of surveillance of the movement behaviours means that this high prevalence of not meeting the guidelines is, in effect, invisible. Lack of visibility leads to policy inaction.^6^ There are marked health inequalities in the outcomes of movement behaviours by early childhood (e.g., obesity), and interventions to reduce health inequalities are generally more (cost-)effective when implemented early in life.^7^

Box 1 **Three tests to examine if European public health policy and clinical practice were sufficiently responsive to the WHO Guidelines.**

**Table:** 

**Test 1: newly created national guidelines or adoption/adaptation of the WHO Guidelines**
Adoption/adaptation of the WHO Guidelines has taken place in several countries outside Europe. Guideline development/adoption groups in those countries considered guidelines to be fundamental to policy formation and implementation, and to achieving impact in clinical practice and public health. Among the 13 countries represented by the SUNRISE Study Europe Group, only two (Finland and Portugal) currently have national guidelines for PA, screen-time and sleep in the under-5s (Supplementary Figure S2). In Belgium, only the Flanders region has guidelines in place. Overall, *European countries have failed Test 1.*
**Test 2: surveillance of the movement behaviours**
Surveillance of health behaviours is a pillar of public health, essential to understand: if guidelines are being met; inequalities and temporal trends; the effects of policies and societal and environmental changes; and how public health resources should be allocated. Historically, surveillance of movement behaviours in children has been limited and–in response to gaps in surveillance in early childhood–the SUNRISE Study was formed.^4^ For this Commentary, one lead expert from each country participating in the SUNRISE Study in Europe reported whether there was national surveillance in their country for all movement behaviours (PA, SB, and sleep) and for all three age groups covered by the WHO guidelines (infants, toddlers, 3- to 4-year-olds). Supplementary Figure S2 shows the considerable gaps in the surveillance of movement behaviours in the under 5-s across Europe. *Test 2 has clearly failed*.
**Test 3: creating national health policy for all movement behaviours.**
The lead experts from each SUNRISE Study Europe Group country were asked “is there specific policy on all of the movement behaviours for the under-5s in your country?”. In all cases (13/13), with the exception of local and regional policies in Flanders, the answer was “no”, *representing a widespread failure of Test 3* (Supplementary Figure S2).

The SUNRISE Study Europe Group proposes that countries across Europe take actions within 5 years (Fig. 1). PA policymaking for school-age children and adolescents is reasonably good across Europe and many countries in the SUNRISE Study Europe Group have PA guidelines for the under-5s (Supplementary File), as do approximately one-third of countries in the WHO EURO region.^8^ Policymaking on PA itself is however insufficient and should extend to sedentary behaviour, including screen time, and sleep.^2^ Additionally, sufficient policy implementation and evaluation—which are often lacking—is required. Policy implementation should also encompass ‘upstream’ influences on the movement behaviours. Behaviours are heavily influenced by the environment in its widest sense (physical, built, social-cultural and policy environments) and for the under-5s early childhood education and care environments are particularly important. Effective policies targeted at upstream environmental influences will be required to equitably change the movement behaviours across European populations.

**Fig. 1:**
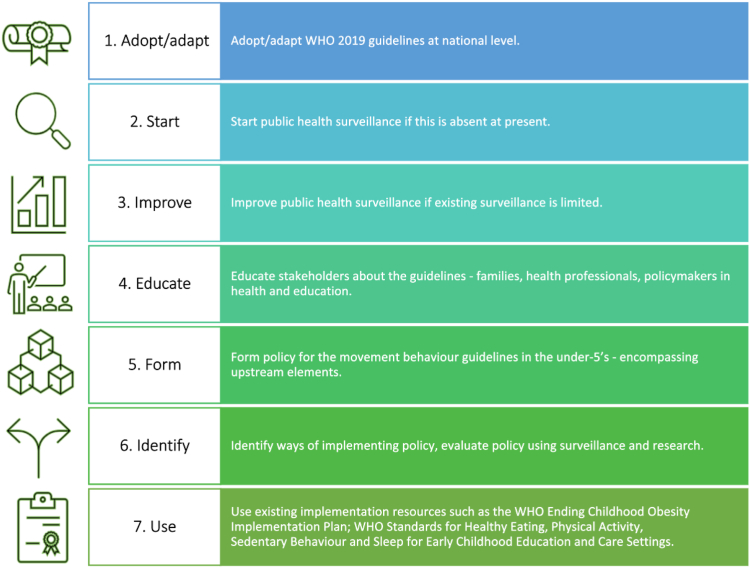
Practical steps needed to achieve impact of the WHO 2019 movement behaviour guidelines for the under-5's across Europe from 2024 to 2029.

There are some grounds for optimism. In contrast to many other health behaviours at other stages of the lifecourse, the movement behaviours in early life can be improved substantially with evidence-based interventions in policy and practice. Resources to help stakeholders improve the movement behaviours are available, and there is preliminary evidence that these can be successful, with promising case studies that might be useful models for wider implementation.^9^ Having guidelines encourages the development of surveillance, which in turn prompts policy action when public health problems become visible.^6^

As representatives of the SUNRISE Study Europe Group, and authors of a number of evidence-based movement behaviour guidelines and strategies nationally and internationally, it is suggested that the steps summarised in Figure 1 are taken across Europe. The steps needed will have short-term costs, but such costs have clearly not been a major barrier to those countries outside Europe, that have adopted or adapted guidelines for all of the movement behaviours in infants, toddlers, and 3- to 4-year-olds and used these as the basis for surveillance and policymaking. Launching the Guidelines in 2019, the WHO Director-General Dr Tedros Adhanom Ghebreyesus said that “Early childhood is a period of rapid development when family lifestyles can be adapted to boost health gains”. Healthier movement behaviours in early life will not only improve health outcomes, but also contribute to achieving many of the Sustainable Development Goals via co-benefits for educational outcomes, the economy and planetary health.^10^

## Contributors

Conceptualization: J.J.R, S.L.C.V, M.D.C.; Methodology: J.J.R, S.L.C.V, M.D.C.; Supervision: J.J.R.; Visualization: H.N.; Writing—original draft: J.J.R.; Writing—review & editing: M.D.C., S.L.C.V., L.B.A., F.B., J.D.P.C., E.E., J.J., A.K., M.L., C.M., H.N., A.O., M.T., F.V., M.Y., J.J.R.

## Editor note

The Lancet Group takes a neutral position with respect to territorial claims in published maps and institutional affiliations.

## Declaration of interests

The authors have no competing interests to declare.
