# Supplementary material for: Practical steps needed to achieve impact of the WHO 2019 movement behaviour guidelines for children under the age of 5: the SUNRISE Study Europe Group evaluation
Source: Lancet Reg Health Eur. 2024 Feb 28;39:100869. doi: 10.1016/j.lanepe.2024.100869 (PMC11129333; doi:10.1016/j.lanepe.2024.100869)
Supplement: Supplementary Figures S1 and S2 and Supplementary Table S1 [file mmc1.docx]

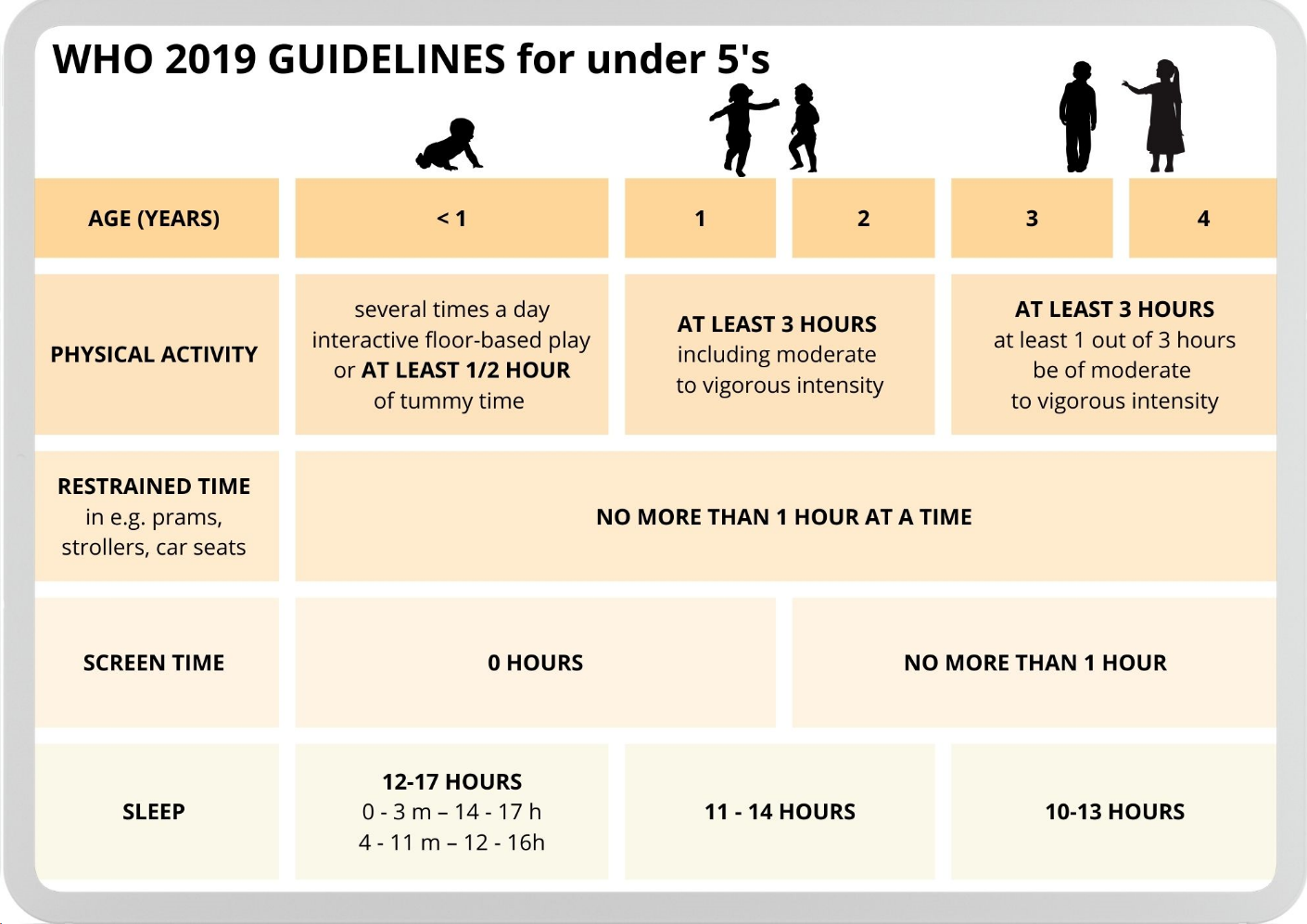
 **Supplementary** **Figure 1.** The WHO 2019 Guidelines for the 24-hour movement behaviours for the under-5s^2^.


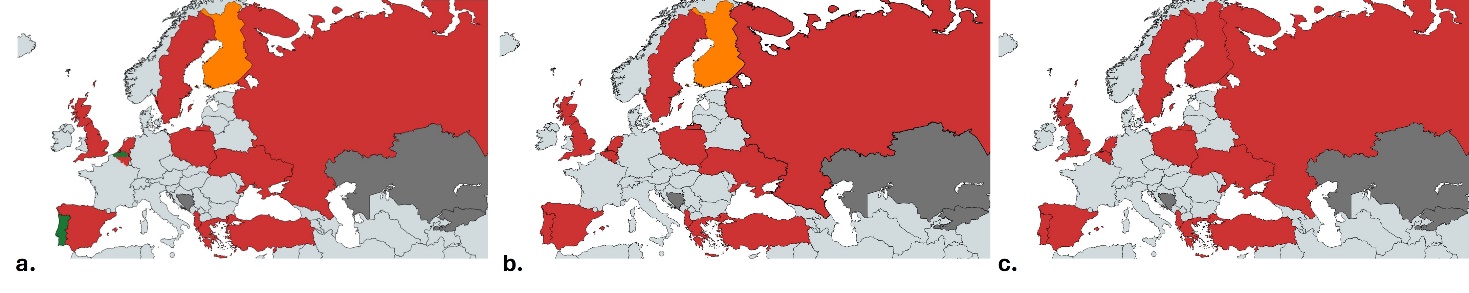
**Supplementary** **Figure 2.** Three tests summarising impact of the WHO movement behaviour guidelines in the 11 SUNRISE countries in Europe for all three behaviours ( physical activity, sedentary behaviour and sleep) in all three age groups (infants, toddlers, and 3-4 year olds): **a. Test 1:** Adoption/adaptation of the WHO Movement Behaviour Guidelines; **b. Test 2:** Surveillance of the movement behaviours **c. Test 3:** Creation of specific national health policy. COLORS CODING: green - YES, amber – SOME/PARTIAL, red – NO, dark grey – Sunrise country with no data

**Supplementary Table 1: Physical Activity/Sedentary Behaviour/Sleep Guidelines, Surveillance, and Policy for infants, toddlers and 3- to 4-year-olds from 13 nations in the SUNRISE Study Europe Group.**

| **Country** | **National guidelines for time spent in all behaviours, all three age groups** | **National surveillance system for time spent in all behaviours, all three age groups** | **Specific health policy for time spent all behaviours, all three age groups** | **Comments** |
| --- | --- | --- | --- | --- |
| Albania | No | No | No |  |
| Belgium | Flanders only | No | No |  |
| England | No. Physical activity only | No, physical activity only and not for all three age groups | No |  |
| Finland | Yes, but same guideline for all children under school-age, not specific to infants and toddlers | Yes, but for 4- to 6-year-olds only | No | Amber in Fig.1 Test 1 and 2 because of lack of age-specificity of guidance and surveillance for all age groups |
| Greece | No | No | No |  |
| Netherlands | Partial. Physical activity and sedentary behaviour only. | No, physical activity only | No | Surveillance will start in 2025 |
| Poland | No. | No | No | Surveillance will start in 2025 |
| Portugal | Yes | No | No |  |
| Russian Federation | No. Physical activity only | No. Physical activity only, not for all age groups | No |  |
| Scotland | No. Physical activity only | No. Physical activity only, not for all age groups | No |  |
| Spain | No | No | No |  |
| Sweden | No. Physical activity only. | No | No | Guidance for some behaviours for some age groups, but not comprehensive and/or time-specific |
| Turkey | No | No | No |  |

Ukraine is a member of the SUNRISE Study Europe Group, but participation in this Commentary was not possible because of the involvement of Russia. In Ukraine responses to the WHO 2019 Guidelines have been disrupted and delayed by the Russian invasion.
